# Impact of Long-Term Alkaline Cleaning on Ultrafiltration Tubular PVDF Membrane Performances

**DOI:** 10.3390/membranes15070192

**Published:** 2025-06-27

**Authors:** Marek Gryta, Piotr Woźniak

**Affiliations:** Faculty of Chemical Technology and Engineering, West Pomeranian University of Technology in Szczecin, Piastów Ave. 17, 70-310 Szczecin, Poland; piotr.wozniak@zut.edu.pl

**Keywords:** car wash wastewater, ultrafiltration, fouling, alkaline cleaning, PVDF membrane, membrane endurance

## Abstract

The application of an ultrafiltration (UF) process with periodic membrane cleaning with the use of alkaline detergent solutions was proposed for the recovery of wash water from car wash effluent. In order to test the resistance of the membranes to the degradation caused by the cleaning solutions, a pilot plant study was carried out for almost two years. The installation included an industrial module with FP100 tubular membranes made of polyvinylidene fluoride (PVDF). The module was fed with synthetic effluent obtained by mixing foaming agents and hydrowax. To limit the fouling phenomenon, the membranes were cleaned cyclically with P3 Ultrasil 11 solution (pH = 11.7) or Insect solution (pH = 11.5). During plant shutdowns, the membrane module was maintained with a sodium metabisulphite solution. Changes in the permeate flux, turbidity, COD, and surfactant rejection were analysed during the study. Scanning electron microscopy (SEM), atomic force microscopy (AFM), differential scanning calorimetry (DSC), and Fourier-transform infrared spectroscopy (FTIR) analysis were used to determine the changes in the membrane structure. As a result of the repeated chemical cleaning, the pore size increased, resulting in a more than 50% increase in permeate flux. However, the quality of the recovered wash water did not deteriorate, as an additional separation layer was formed on the membrane surface due to the fouling phenomenon.

## 1. Introduction

Washing cars requires large amounts of water; thus, many car washes use water recycling systems [1,2]. Today, more than 50% of car washes have recycling systems that use sedimentation and sand filtration supported by water aeration [3,4,5]. In addition, membrane recycling systems are increasingly being developed [6,7,8,9].

The applied type of wastewater treatment system strongly depends on the size of the car wash. Large automated car washes use multi-stage systems that produce high-quality wash water, but this is expensive [3]. The average car wash customer spends $15 per visit, and the global car wash market is estimated to be worth $41 billion by 2025 [2]. Such revenues allow for investment, and 85% of professional car washes use water reclamation systems [10].

The financial situation is different for small car washes. Such car washes are generally supplied with municipal water and discharge their effluent into the sewerage system. In Poland, car wash owners pay $5–7 per 1 m^3^ of water for such use. Such low prices do not provide an incentive to invest in expensive equipment to reduce fresh water consumption. Most of these car washes have a reverse osmosis (RO) system (pump, filter, and membrane module) that produces pure water, resulting in a spot-free final car rinse. Owners of such car washes expect a similarly simple ultrafiltration (UF) system to be used for water recycling. The development of this solution is the focus of the research presented here. Such a simple system for recovering wash water has been proposed using ultrafiltration tubular polyvinylidene fluoride (PVDF) membranes [11]. PVDF membranes are commonly used in the manufacture of industrial microfiltration and ultrafiltration modules [12,13]. However, car wash wastewater treatment by UF causes the rapid fouling of the membranes [3,7,14,15].

It is well-known that the fouling phenomenon is a fundamental obstacle to the industrial application of membrane processes [16,17]. The fouling layer formed on the membrane surface can be removed by physical methods such as backwashing or flushing [18]. Unfortunately, such methods are often insufficient for wastewater separation and, consequently, the chemical cleaning of the membrane is required [12,19]. Cleaning agents containing sodium hydroxide (NaOH) and sodium hypochlorite (NaClO_3_) are used for membrane washing [12,13]. Such solutions are aggressive, and, therefore, their frequent and prolonged application causes damage of the polymeric membranes [12,20,21,22]. However, alkaline detergents, such as P3 Ultrasil 11, are commonly used in membrane plants due to their high cleaning efficiency [13,23,24,25]. To limit membrane damage, companies generally specify their use [13,26]. However, for new technologies, it is necessary to test the resistance of the membranes to such detergents. The results of studies involving filtration for a maximum of a few days are mostly presented, and only a few papers present longer studies, such as 120-day tests of PVDF membranes [27]. Short-term tests do not provide satisfactory information; hence, in this study, tests lasting several months were carried out.

The popular spiral wound UF modules cannot be used for car wash wastewater separation due to the high turbidity [3,14]. Their application requires the use of a pre-filtration stage [3,7], which is unfavourable for small car washes as it increases the size (cost) of the treatment system. For these reasons, tubular PVDF membranes have been used to treat car wash wastewater [11,28]. It has been shown that the UF permeate obtained from the filtration of the wastewater can be successfully used for car washing [29]. In this study, PVDF FP100 tubular membranes, installed in the B1 module manufactured by PCI, were successfully used to produce washing water. A permeate flux of 50–60 L/m^2^h was achieved during the UF of real wastewater treatment at a pressure differential of 0.1 MPa [11]. For such a process’ yield, the UF plant consisting of two to five B1 modules would be suitable for small car washes.

One limitation of the presented solution is the considerable fouling caused by the compounds of car wash wastewater. In this case, the fouling of the UF membranes was reduced by membrane washing with cleaning agents containing NaOH, such as P3 Ultrasil 11 and an agent used in car washes to remove insects [11,14,15]. Membranes can also be cleaned with NaClO_3_ solutions; however, these cause much more damage to the PVDF membrane [12]. Previous studies have obtained results for FP100 membranes in tests involving a small module lasting only a few weeks [11]. While these results are promising, they are insufficient to convince washer manufacturers to modify their designs and incorporate UF plants into their washing systems. What is required is proof that the proposed method of cleaning the UF membranes will prevent them from failing quickly. To obtain such data, a two-year study of an industrial B1 module was conducted.

The performed UF studies showed that PVDF membranes are not fully resistant to NaOH solutions. As a result, it has been observed that cyclic filtration and cleaning has altered the stability of PVDF membranes [12,30]. On the other hand, it has been observed that fouling and the presence of surfactants reduce the negative effect of alkaline solutions [31], which may reduce the degradation of PVDF membranes during car wash wastewater separation. However, the degradation process can be very slow and its deleterious effects can significantly damage the membranes after several months of membrane module operation. Following several weeks of testing FP100 membranes, it was found that alkaline cleaning caused minor damage to the membranes’ active layer [11]. This raises the question of whether these membranes could withstand several years of exposure in car washes. Due to the lack of such data, a pilot scale study was undertaken in this work.

For industrial implementation, commercially available membrane modules are required. Hence, the B1 module from PCI with FP100 membranes were chosen for the study. This module has been successfully used for several years in studies on oily wastewater separation [32]. However, the feed solutions tested in this work, such as bilge water, were not alkaline. For this reason, the industrial PVDF membrane module tested in this study was fed with a mixture of car wash chemicals (pH = 8.5–9), which is similar in composition to the wastewater generated at car washes. Moreover, the membranes were cleaned cyclically using alkaline agents with pH 11–12. During shutdowns, the membrane module was maintained with a sodium metabisulfite solution. The effect of the repeated chemical cleaning of fouled PVDF membranes on the permeate flux and surfactant rejection was analysed. Scanning electron microscopy (SEM), atomic force microscopy (AFM), differential scanning calorimetry (DSC) and Fourier-transform infrared spectroscopy (FTIR) analysis were used to analyse the changes in the membrane structure. The main objective of the study was to determine whether the selected tubular module would remain undamaged over the long term and whether the quality of the permeate obtained would not deteriorate significantly.

## 2. Materials and Methods

### 2.1. UF Installation

**Figure 1:**
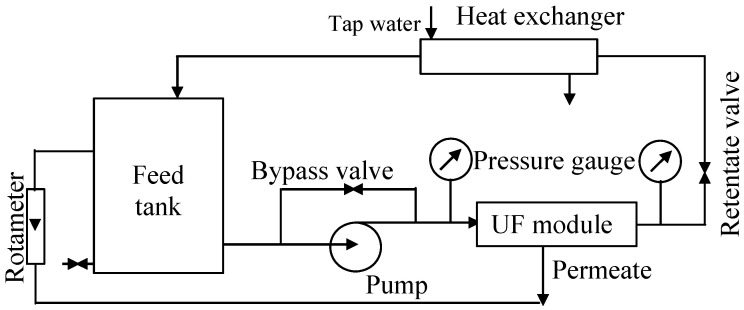
Experimental set-up of the pilot-scale UF unit.

The UF process was investigated using the UF pilot plant shown schematically in Figure 1. The unit was equipped with a PCI B1 tubular module with 18 FP100 (100 kDa) polyvinylidene fluoride membranes (PCI Membranes, Kostrzyn Wielkopolski, Poland) mounted inside (Table 1). The diameter and length of the tubular membrane were 1.25 cm and 120 cm, respectively. The feed tank (200 L) was connected to a VNR-8 centrifugal pump (Grundfos, Bjerringbro, Denmark). The feed flowed inside the tubular membranes at a velocity of 1.9 m/s, and the UF tests were performed at a transmembrane pressure (TMP) of 0.1 MPa. For all types of solutions tested, the feed temperature was 298 ± 2 K. Temperature control was provided by a tubular heat exchanger (cooled by tap water), through which the feed flowed. The UF tests were carried out for 7–8 h a day, with the feed remaining in the system overnight. Overnight breaks also occur in most car washes, as 85% of car wash customers prefer to visit car washes during daylight hours [10].

The permeate flux [L/m^2^h] was obtained based on the following equation:

(1) $$J=\frac{V}{S\cdot t}$$

where V [L]—cumulative permeate volume; S [m^2^]—effective membrane surface area; and t [h]—filtration duration.

The rejection efficiency R [%] was determined as follows:

(2) $$R=1-\frac{C_{p}}{C_{F}}\cdot 100\%$$

where C_P_ [mg/L] and C_F_ [mg/L] are the measured concentrations of the permeate and feed, respectively.

The UF tests were conducted at constant feed concentrations, with the permeate returned to the tank. Initially, the feed tank was filled with 100 L of the test solutions.

### 2.2. Feed Solutions

The permeate from the nanofiltration (NF) process, fed with tap water, was used to flush/rinsing the UF unit and to prepare test solutions. The NF permeate obtained had a conductivity in the range of 70–90 μS/cm and its composition has been reported in previous work [32,33].

In car washes, cars are first washed with various foam solutions and, then, after rinsing with water, their surfaces are treated with hydrophobic coatings (hydrowax). Synthetic car wash wastewater has been produced from a mixture of these agents with a composition similar to that of a car wash. Detergents (Euro Foams and Hydrowax) produced by EuroEcol (Łódź, Poland) were used. The foam solutions contained surfactants, diethylene glycol butyl ether, benzene sulphonic acid, and polymers. The exact composition of the commercial concentrates is given in [14,15]. The mixture used for the UF tests contained 0.5 vol.% foaming agent and 0.2 vol.% Hydrowax solution (EuroEcol, Łódz, Poland). In addition to Euro Turbo Foam without dyes (“White”), foam agent concentrates with dyes were also used: Euro Turbo Foam Colour Green (“Green”) and Euro Turbo Foam Colour Blue (“Blue”). The latter contained indigo carmine dye, which increased the fouling of PVDF membranes [34]. The parameters of the synthetic wastewater are shown in Table 2.

Before weekend shutdowns, the feed was removed, and the plant was flushed with water (200 L) and filled with NF permeate. For longer shutdowns, additional maintenance was performed by flushing the system with 0.25 wt.% sodium metabisulphite (Na_2_S_2_O_5_) solution (ChemLand, Stargard, Poland). This preservative solution was recirculated for 15 min, after which the system was drained. The resumption of UF operation after the break was started by rinsing the UF installations twice with NF permeate (2 × 100 L).

Separation of the wastewater caused fouling and a consequent reduction in permeate flux. The membranes were, therefore, chemically cleaned at regular intervals. PCI company recommends P3 Ultrasil 11 from Hankel Ecolab (Suturamed, Szczecin, Poland) for washing the FP100 membranes. This agent contains surfactants and tetra sodium salt of EDTA in addition to NaOH [23]. A 0.1 wt.% solution with pH = 11.7 was prepared for washing the UF plant. A similar pH value is found in the 0.5 vol.% solution of Insect (EuroEcol, Łódź, Poland). This detergent contains NaOH and is used in commercial car washes to remove insects from car bodies. It has also been shown to be effective in removing deposits formed on membranes during the filtration of car wash wastewater [11,14]. The prepared cleaning solutions (100 L) were recirculated for 30 min (TMP = 0.02 MPa). After chemical cleaning, the UF system was flushed twice with NF permeate (100 L) and the maximum permeate flux was determined (TMP = 0.1 MPa).

### 2.3. Analytical

The Hach cuvette tests were used to determine the concentration of surfactants (LCK 333—nonionic, and LCK 432—anionic) and chemical oxygen demand—COD (LCK 1014). The values were recorded automatically with the use of a Hach Lange DR2800 spectrophotometer (Hach Lange, Düsseldorf, Germany).

The membrane surface was examined by atomic force microscopy. A multi-Mode 8 AFM apparatus equipped with a Nanoscope V converter from Bruker (Billerica, MA, USA) characterised the membrane roughness in the scanasyst mode. The Ra and Rq parameters were evaluated on a basis of at least five AFM images (10 μm × 10 μm).

The turbidity of tested solutions was measured with a portable turbidity meter model 2100 AN IS with a detection limit of 0.01 NTU (Hach Company, Loveland, CO, USA).

Differential scanning calorimetry analysis (DSC) was performed in a NETZSCH STA 449 F3 Jupiter (Erich NETZSCH GmbH & Co. Holding KG, Selb, Germany). The samples were heated from room temperature to 523 K at a heating rate of 2 K/min.

## 3. Results and Discussion

### 3.1. Car Wash Wastewater Ultrafiltration

**Figure 2:**
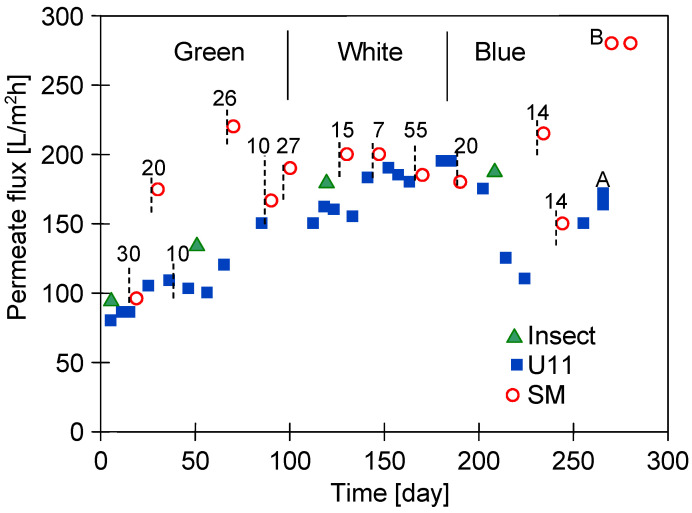
Changes in the maximum permeate flux during UF pilot plant exploitation. NF permeate used as a feed. Flux determined after membranes cleaning. Cleaning solutions: Insect—0.5 vol.% of Insect agent concentrate (pH = 11.5), U11—0.1 wt.% P3 Ultrasil 11 (pH = 11.7), and SM—0.25 wt.% sodium metabisulfite. Numbers—days of shutdowns periods. Point A—membranes washed with hot 0.1 wt.% P3 Ultrasil 11 solution. Point B—installation rinsed with 0.25 wt.% sodium metabisulfite solution.

The PVDF module installed in the UF plant was tested for almost two years, separating different types of car wash wastewater (Green, White, and Blue—Table 2). Changes in the maximum permeate flux after chemical cleaning are shown in Figure 2. The installation operated for five days a week, totalling almost 300 working days in the study. In addition to the weekend breaks, there were also several 7- to 55-day shutdowns periods, before which the installation was maintained with a sodium metabisulfite solution. It is worth noting that a similar situation can occur with small car washes, as their owners also do not work on holidays. Figure 2 shows the numbers indicating longer shutdown periods.

As shown in Figure 2, repeated washing of the membrane with NaOH solutions increased the permeate flux, possibly due to an increase in the membranes’ pore size [12]. The largest changes occurred during the initial 40 days’ (excluding shutdowns) exploitation period, which was also observed in other pilot studies [13]. After this period, the maximum permeate flux increased from 95 to 175 L/m^2^h. Subsequent to the third maintenance of the plant with an Na_2_S_2_O_5_ solution, the permeate flux increased to 220 L/m^2^h. Lower maximum permeate flux values were obtained after washing the membrane with a P3 Ultrasil 11 solution. The conducted membrane washing with this agent probably did not remove all contaminants, especially those in the largest pores [11]. These pores were cleaned to a greater extent by the sodium metabisulphite solution and, as a result, the highest values of maximum permeate flux were obtained after plant shutdowns (points SM). It has been found that, after one year of module operation (Figure 2, after 140 days), this effect disappeared. This could be due to a better cleaning of the fouling layer (generated during White wastewater separation) also by the P3 Ultrasil 11 solution. Differences again occurred in the separation of Blue wastewater, as it contained components that were more difficult to remove by alkaline cleaning solutions. These wastewaters were found to contain indigo carmine dye, which was found to adsorb strongly onto the surface of PVDF membranes [34].

A higher flux recovery for membranes contaminated with Blue wastewater was achieved by washing the module twice (for 30 min each time) with a hot (50 °C) solution of P3 Ultrasill 11 (Figure 2, point A), after which the UF plant was rinsed with a sodium metabisulphite solution. Consequently, the maximum permeate flux increased to 275 L/m^2^h (point B). This indicates that this procedure allowed us to clean the membranes and led to an increase in their pore size.

After one week of ultrafiltration testing, the plant was rinsed again with a sodium metabisulphite solution; however, this did not result in any further increase in the permeate flux. This suggests that the Na_2_S_2_O_5_ solution did not enlarge the pores of the tested membranes, but, rather, cleaned them thoroughly. While the membrane manufacturer permits the use of hot P3 Ultrasil 11 solution for cleaning purposes, it should be noted that this will accelerate the adverse effects of NaOH on the PVDF membrane matrix.

**Figure 3:**
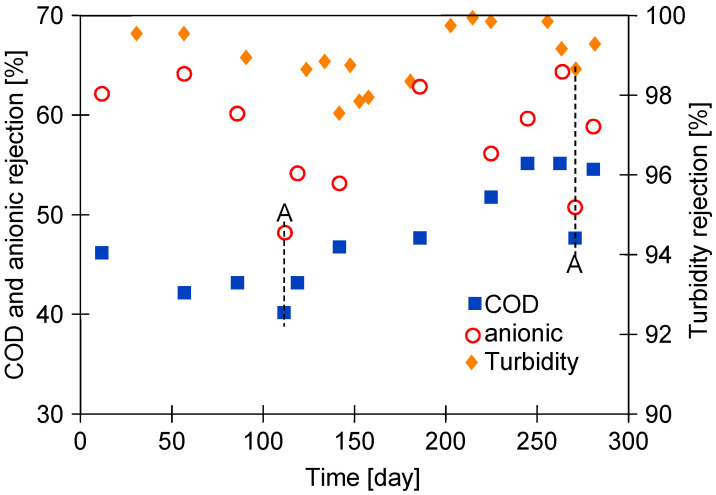
Changes in rejection of COD, turbidity, and anionic surfactants during UF studies (samples collected after one-day UF of wastewater). Point A—parameter determined direct after membrane cleaning (cleaning solutions: 0.25 wt.% sodium metabisulfite).

Obviously, increasing the pore size increases permeate flux; however, this also worsens rejection [35]. However, in ongoing UF studies, it is important to note that the results presented in the current study demonstrated that no significant change in rejection (Figure 3) was observed despite the increase in permeate flux (Figure 2). In addition, the separation of the Blue wastewater improved the rejection (Figure 3, after 200 days). The Blue wastewater had a significantly higher turbidity (Table 2). This increases the formation of a filter cake, which acts as an additional separation layer and, consequently, improves the separation [36]. Chemical cleaning removes most of the filter cake from the membrane surface, and, as a result, a deterioration in rejection was observed (Figure 3, points A).

**Figure 4:**
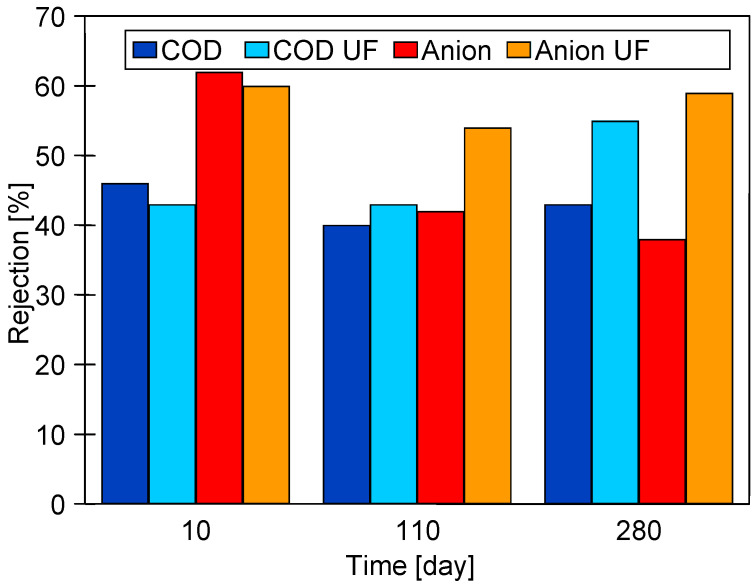
Changes in the rejection during performed UF studies. Membranes after chemical cleaning (COD and Anion) and after 2–3 days of UF process covered by fouling layer (COD UF and Anion UF).

The positive influence formation of the fouling layer on the rejection is also confirmed by the results shown in Figure 4. The separation results obtained immediately after membrane chemical cleaning (COD and Anion) and the results after 2–3 days of wastewater filtration (COD UF and Anion UF) can be seen. The results obtained after chemical cleaning of the membrane deteriorate with the time of filtration of the UF module, especially for the removal of anionic surfactants, can also be seen. This finding confirms that the repeated chemical cleaning of the membrane with alkaline agents caused an increase in pore size. However, during the filtration of the effluents, due to fouling, the separation improves and remains at a similar level throughout the UF test period.

**Figure 5:**
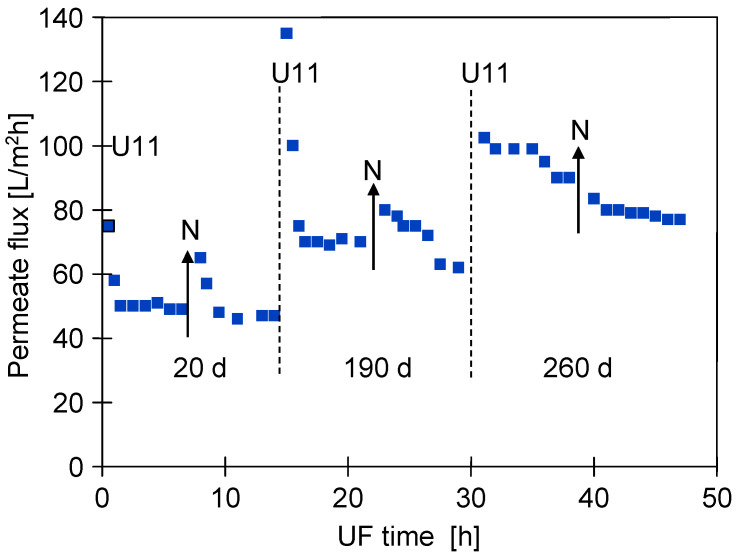
Effect of UF module lifetime (d—days) on permeate flux changes during car wash wastewater separation. Points: U11—membranes washed with P3 Ultrasil 11; N—overnight stops, installation filled with NF permeate.

The changes in the membrane structure induced by the chemical cleaning also affected the filtration of the car wash wastewater. Figure 5 shows an example of the changes in permeate flux obtained during two consecutive days of UF testing at different times of the module exploitation. During the initial period for the new membranes, the flux decreased rapidly and stabilised at 50 L/m^2^h. Similar 50% permeate flux decreases after 60 min of UF have been reported in other work [7,11]. The repeated cleaning of the membranes with NaOH solutions resulted in an increase in membrane permeability (Figure 2) and, consequently, an increase in permeate flux during wastewater separation.

In addition, the permeate flux also increased after overnight stops (Figure 5, point N). This was due to the decompression of the membrane and sediment (TMP = 0) and, also, to the phenomenon of osmotic washing (module filled with NF permeate). The pore size increased with the time of use and this effect had practically disappeared after one year of operation (Figure 5, after 30 h). Importantly, the larger pores favour internal fouling, which is more difficult to wash off during overnight soaking. Therefore, an increase in permeate flux was only achieved after intensive chemical cleaning (Figure 2, after 250 h). It was found that rinsing the system with sodium metabisulphite solution not only maintained the membranes but also resulted in significant cleaning. As a result, a significant increase in permeate flux was observed after each system maintenance (Figure 2, SM).

### 3.2. Membrane Morphology

**Figure 6:**
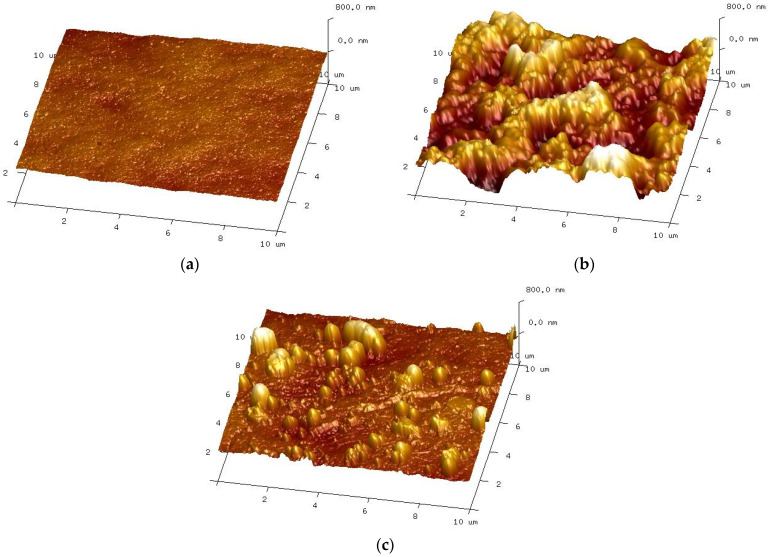
AFM images of the membranes surface: (**a**) new membrane; (**b**) membrane used for separation wastewater; and (**c**) membrane after HCl rinsing.

On completion of the UF tests, the membrane was removed from the module and rinsed with deionised water. The membrane surface was brown in colour, indicating that iron oxides were also present in the resulting fouling layer [32]. After washing with a 30 wt.% HCl solution, the membranes regained their white colour. The AFM images of the surface of such washed membranes and the new membrane are shown in Figure 6.

The AFM analysis confirmed that the membrane surface changed during the wastewater separation and cyclic membrane washing with the alkaline detergents. Despite intensive washing, large amounts of contaminants remained on the membrane surface (Figure 6b). HCl rinsing removed most of these sediments, but the sediment residues are still visible on the membrane surface (Figure 6c). Due to the formation of deposits, the roughness parameters for the tested membranes increased significantly during the UF of car wash wastewater. The values of Rq increased from 35.1 to 223.7 nm, and Ra from 28.2 to 179.2 nm (Table 3, after UF tests). The roughness parameters after rinsing with HCl solution decreased, e.g., for Rq, from 223.7 to 114.5 nm; however, these were still significantly higher than the values set for the new membranes.

The AFM images show that the sediment structure was not dense and that the numerous voids visible facilitated the transport of the filtrate to the membrane surface. As a result, the formation of a filter cake of considerable thickness did not lead to a decrease in permeate flux (Figure 5).

**Figure 7:**
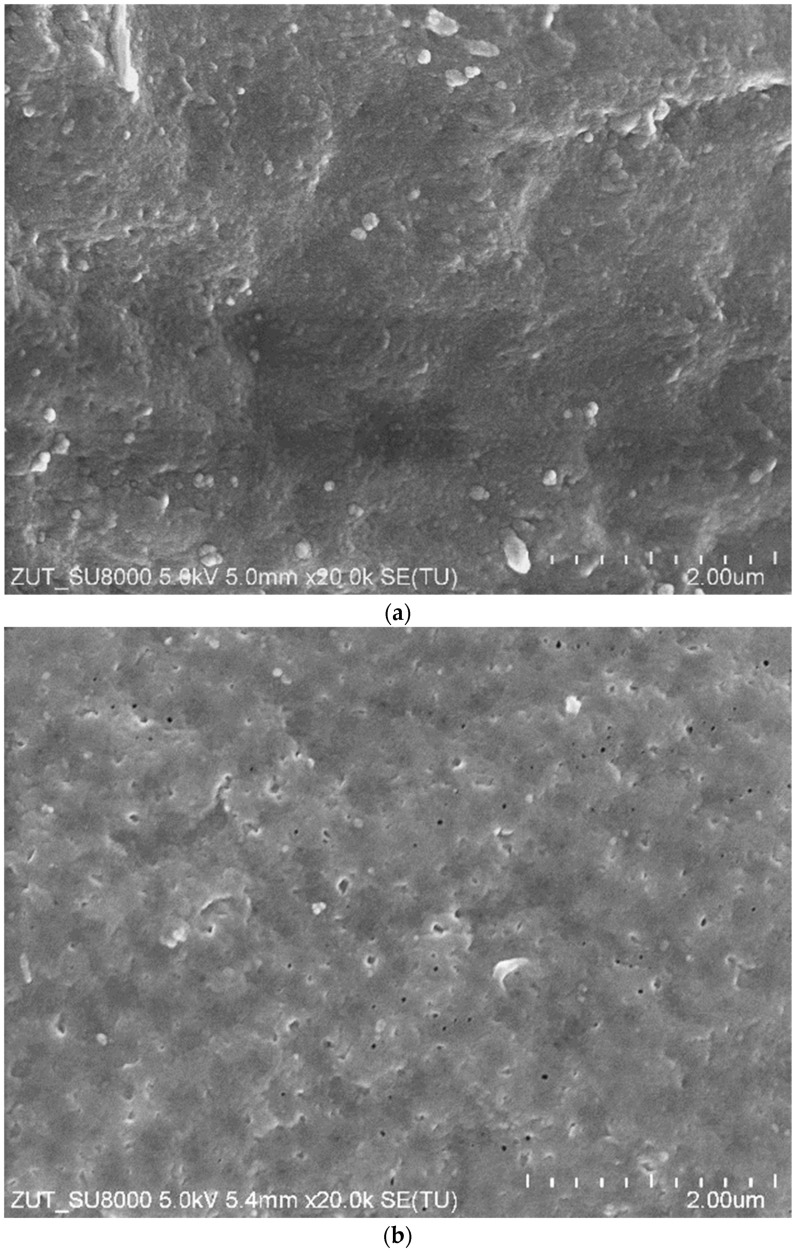
SEM images of FP100 membrane surface: (**a**) new membrane, and (**b**) membrane after 2 years of UF tests.

The areas of the membrane surface exposed after washing with HCl solution show no damage (Figure 6c), and, in these areas, the image is similar to that obtained for a new membrane (Figure 6a). However, the SEM observation revealed that, after the UF tests, the number of larger pores on the surface of the active layer increased compared to the new membrane (Figure 7). Consequently, the membrane’s permeability increased, resulting in an increase in the permeate flux over the course of the module operation (Figure 2 and Figure 5). The obtained SEM results indicate that the repeated cleaning of the membrane with an alkaline agent can cause minor changes to the active layer’s structure. This also confirms that PVDF membranes are not fully resistant to the prolonged exposure to NaOH solutions.

These results raise the question of whether the proposed alkaline washing method would damage the PVDF membranes to such an extent that they could not be used to obtain wash water from car wash wastewater. As demonstrated in Figure 3 and Figure 4, the thorough cleaning of the pores worsens the retention of wastewater constituents. However, once wastewater filtration begins, these pores quickly become blocked by sludge (Figure 6b), which improves the rejection degree (Figure 4). Consequently, despite the formation of larger pores, the tested membranes still effectively filter out turbidity (Figure 3) and bacteria [11], and the UF permeate obtained from the wastewater was successfully used for car washing [29].

**Figure 8:**
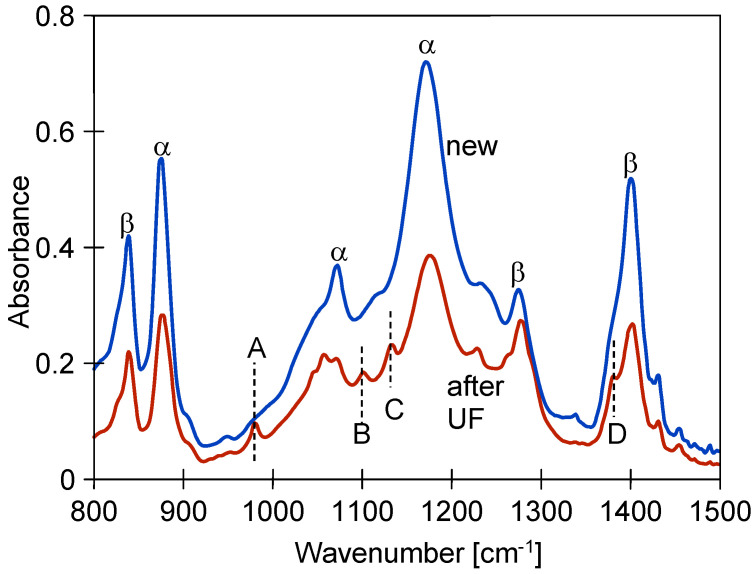
FTIR analysis of new FP100 and after UF test membrane samples.

In addition to enlarging the pore size, the long-term use could have caused changes to the matrix structure of the PVDF membranes. For example, this could result in membrane cracking. PVDF forms five crystalline polymorphs, the most common of which are the α and β forms, which affect membrane performance differently [37]. Transitioning from the β to α phase can negatively impact the membrane’s flexibility [37,38]. The FTIR spectra of the studied membranes revealed several absorption bands that can be attributed to the α and β conformations of PVDF (Figure 8).

Characteristic bands appeared at 875, 976, 1070, and 1181 cm^−1^, indicating the α conformation, while the 841, 1279, and 1403 cm^−1^ results belong to the β phase [12,37,38]. An FTIR analysis showed that the intensity of these peaks decreased significantly as a result of the UF tests (Figure 8). New peaks also appeared in the membranes after two years of testing and are labelled A, B, C, and D in Figure 8. Peak A, at 978 cm^−1^ (assigned to CH_2_ twisting), is attributed to the α-conformation [12]. Similarly, peak D (1383 cm^−1^) indicates the occurrence of phase α [37]. However, the intensity of these peaks is low, suggesting that a significant conversion of phase β to the α conformation does not occur in the studied membranes.

**Figure 9:**
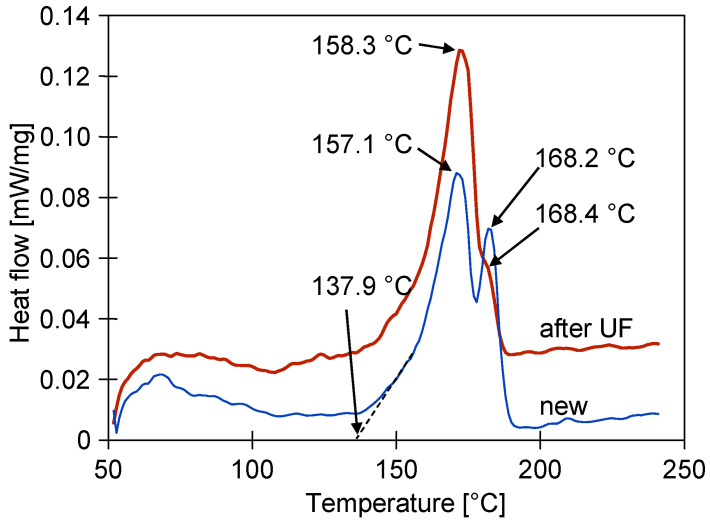
DSC analysis of new FP100 membrane sample, and membrane sample collected from module after two years of UF tests.

The changes in the structure of the PVDF membranes can be revealed by the melting point and enthalpy of the phase transformation measurements in the DSC analysis [39]. The DSC signal of the new membrane shows an endothermic effect (peak temperatures of 157.1 °C and 168.2 °C) starting from an extrapolated initial temperature of 137.9 °C (Figure 9). The endothermic DSC peaks are due to the melting of the sample and the calculated enthalpy was 53.05 J/g. The results obtained for the sample after the UF tests showed that the second peak decreased, while the peak temperatures were 158.3 °C and 168.4 °C for the first and second peaks, respectively. In this case, the enthalpy decreased from 53.05 to 50.75 J/g. All of the determined melting temperatures are below 179 °C, indicating that phase α and β are the only phases of PVDF present in the studied samples [37].

Moreover, the results obtained indicate that the almost two years of the module operation induced some changes in the polymeric structure of the PVDF membranes studied. This finding confirms the results obtained from the FTIR analysis. However, the small differences in the determined melting temperatures indicate that there is little degradation of the membrane matrix.

The performed SEM and AFM examination of the samples after the UF test did not reveal any cracks in the membrane and the rejection value during wastewater separation was close to the initial value (Figure 4); thus, it can be assumed that the membranes tested retained good separation properties despite some changes in the polymeric structure.

## 4. Conclusions

UF tests conducted over a period of almost two years showed that tubular PVDF membranes could be successfully used to separate car wash wastewater of varying composition and turbidity close to 100 NTU. However, filtration of the wastewater caused severe fouling, resulting in a decrease in permeate flux of more than 50% after 2–3 h of UF operation.

The intensity of the fouling phenomenon was reduced by cyclic membrane washing with detergent agents containing NaOH (pH = 11.5–11.7). Satisfactory cleaning efficiency was also demonstrated by a 0.25 wt.% sodium metabisulphite solution, which was used to maintain the UF module during plant shutdown periods.

For a car wash installation with UF, the membrane cleaning programme would include washing the FP100 membrane with a 0.1 wt.% P3 Ultrasil 11 solution. This should be performed at least every five days for 30 min. Additionally, it is recommended to flush the installation with a solution of 0.25 wt.% sodium metabisulphite every fortnight.

After one year of module exploitation with cyclical alkaline washing, the PVDF membranes showed a deterioration in the degree of separation and an increase in permeate flux, indicating that the alkali caused partial pore growth. However, after two years of membrane exploitation, the performed AFM and SEM studies showed no significant damage, although FTIR and DSC studies revealed some changes in the membrane matrix structure. This finding confirms that NaOH solutions have a negative effect on PVDF membranes. However, the resulting changes in the membrane structure do not significantly worsen membrane rejection during wastewater separation. This is due to the fact that the resulting filter cake reduces permeate flux but, at the same time, improves separation.

The results of the current study indicated that periodic cleaning of FP100 membranes (used in car washes) with alkaline agents enables the B1 tubular module to be successfully used for recycling wash water in car washes.

## Figures and Tables

**Table 1:** Tubular module and FP100 membrane characteristics.

Parameter	Unit	B1 Module	FP100
Length	mm	1220	1200
Diameter	mm	100	1.25
Tube	-	18	1
Material	-	AISI 316 Stainless Steel	PVDF
Operating pressure	bar	64	10
Operating temperature	°C	80	80
Volume shroud-side	L	6.7	-
Volume tube-side	L	2.8	0.147
Nominal retention	kDa	-	100
pH range	-	1.5–12	1.5–12

**Table 2:** Parameters of prepared synthetic wastewater. Anionic and nonionic—surfactants.

Wastewater	COD [mg/L]	Anionic [mg/L]	Nonionic [mg/L]	pH [-]	Cond. [μS/cm]	Turbidity [NTU]
White	2275 ± 378	463 ± 26	25 ± 8	8.5 ± 0.2	155 ± 1	11.3 ± 1.4
Green	2150 ± 70	420 ± 20	21 ± 6	8.8 ± 0.2	162 ± 2	12.5 ± 5.2
Blue	2391 ± 115	393 ± 29	40 ± 14	8.6 ± 0.1	167 ± 2	83.2 ± 14.7

**Table 3:** Membranes’ surface roughness.

Parameter	New	After UF Tests	Rinsed
R_q_ [nm]	35.1 ± 1.4	223.7 ± 12.8	114.5 ± 17.4
R_a_ [nm]	28.2 ± 4.1	179.2 ± 9.4	81.9 ± 18.4

## Data Availability

The original contributions presented in the study are included in the article; further inquiries can be directed to the corresponding author.
